# The field situation heuristics effect in online emergencies: the formation mechanism and differences of audience cognitive bias

**DOI:** 10.3389/fpsyg.2026.1567587

**Published:** 2026-02-24

**Authors:** Peng Liu, Changzheng Yang, Jin Gao

**Affiliations:** 1 School of Medical Information and Engineering, Bengbu Medical University, Bengbu, China; 2 College of Liberal Arts, Journalism & Communication, Ocean University of China, Qingdao, China

**Keywords:** situation heuristics, cognitive bias, emergency events, mechanism, media literacy

## Abstract

**Introduction:**

In the contemporary digital communication environment, online emergencies often trigger cognitive biases among audiences, affecting the health of public opinion ecosystems and potentially threatening social stability. While existing research has largely focused on the manifestations and consequences of cognitive biases, the formation mechanisms, particularly the role of contextual factors in the online environment, remain understudied. This study examines how field situational heuristics influence cognitive biases in online emergencies through the mediating pathways of adaptive expectations and implicit attributions.

**Methods:**

This research integrates field theory and heuristic information processing theory to construct a theoretical framework. Using anchoring heuristics, representativeness heuristics, and availability heuristics as independent variables, cognitive bias as the dependent variable, and adaptive expectations and implicit attributions as mediating variables, data were collected through questionnaires and analyzed using structural equation modeling with AMOS 22.0 statistical software.

**Results:**

The findings reveal that: (1) in online emergencies, anchoring heuristics, representativeness heuristics, and availability heuristics exert a significantly positive influence on cognitive biases, mediated by adaptive expectations and implicit attributions; (2) representativeness heuristics have the greatest impact on cognitive biases, followed by availability heuristics, and finally anchoring heuristics; (3) the effect of contextual heuristics on cognitive biases exhibits significant demographic differences both between and within groups.

**Discussion:**

The findings provide theoretical insights for improving online public opinion governance and enhancing audience media literacy. The study highlights the importance of understanding how situational heuristics shape cognitive outcomes in digital communication environments and offers practical implications for managing information dissemination during online emergencies.

## Introduction

Digital technology has fundamentally transformed information dissemination patterns in contemporary society. The 53rd “Statistical Report on the Development of the Internet in China” reveals that China’s internet users reached 1.092 billion by December 2023, with a penetration rate of 77.5%. This digital landscape has established new channels for public expression while simultaneously creating environments where cognitive biases flourish during online emergencies.

Online emergencies are sudden online incidents that trigger widespread public attention and discussion. These events include public safety crises, corporate misconduct incidents, social conflicts, and government action controversies rapidly spreading across digital platforms. Such emergencies should be distinguished from network public opinion, which refers to the collective attitudes and discussions that form in response to these incidents. When online emergencies occur, they generate network public opinion—the aggregated views of internet users—that often exhibit significant cognitive distortions.

The proliferation of online emergencies presents substantial challenges for social governance and information management. These incidents frequently trigger cognitive biases among digital audiences, leading to information distortion, polarized opinions, and potentially destabilizing social impacts. Understanding the mechanisms behind these cognitive biases has become crucial for effectively managing online public discourse.

The rapid development of information technology has continuously introduced new media forms that penetrate every aspect of human life at an unprecedented pace. These technologies have become primary channels for information acquisition and dissemination. Simultaneously, as China’s comprehensive reforms deepen, social development has entered a critical phase characterized by complex social risks and contradictions. With heightened awareness of citizens’ rights, crisis events have become more frequent, emerging as common phenomena in social development processes.

Field theory provides a valuable theoretical foundation for examining these phenomena, emphasizing that the field itself influences individual behavior within a field (McCombs and Valenzuela, 2020). Context is a crucial factor influencing event occurrence and individual behavior within these fields. As Dey (2001) note, context encompasses all information about subject states and environmental changes, including information about entities and surroundings. In online environments, humans are significantly influenced by these contextual factors during social interactions.

Bronfenbrenner (1977) demonstrate that macro environments influence human psychology, behavior, and attitudes specifically through contextual mechanisms. The elaboration likelihood model further clarifies that contextual information primarily affects subject cognition through heuristic information processing pathways (Petty and Cacioppo, 1986). In online emergencies, these contextual heuristics become critical information processing mechanisms through which public opinion fields influence audience cognition.

Current research on cognitive biases in network environments can be systematically categorized into three primary domains:

First, research examining the fundamental characteristics of online public opinion biases has established several key findings. Nazari et al. (2020) identified that online public opinion fundamentally represents allocating netizens’ attention resources and results from non-cooperative games between the public and various stakeholders. Paulus et al. (2024) demonstrated that selective information presentation constitutes a typical cognitive bias arising from subjective thinking, intrinsic motivation, and external environmental factors. Regarding bias characteristics, Bacaksizlar Turbic and Galesic (2023) found that online public opinion biases exhibit diversity and group uncontrollability, while Van Haeringen et al. (2023) identified multi-level derivativeness and guidable controllability that influence collective social emotions and group behaviors. Cai et al. (2023) further characterized these biases by their rapid spread, widespread influence, irrationality, and immediacy.

Second, scholars have investigated the motivational factors and evolutionary processes of online public opinion biases. Brady et al. (2023) established that public emotional tendencies accelerate bias formation processes. Lazer et al. (2018) identified insufficient media responsibility and inadequate accountability mechanisms as critical factors generating biases in online public opinion. Liu et al. (2023) categorized the primary influencing factors as netizens, media, involved parties, opinion leaders, and governmental authorities. Hayes (2022) demonstrated how user psychological factors—including psychological structure, behavioral tendencies, risk perception, and trust structures—significantly affect online public opinion diffusion during emergencies.

Third, research has explored prevention and governance strategies for online public opinion biases. Zhang et al. (2020) emphasized the importance of information collection, crisis early warning, control mechanisms, and transparent information release. Peralta et al. (2021) demonstrated how scientific adjustment of public opinion subjects, rule mechanisms, and relationship structures can inhibit bias formation. Tian et al. (2015) established effective approaches, including rumor spreader isolation, environmental transparency improvement, and persuasion of wavering neutrals. Hallin and Mancini (2004) proposed rectification strategies addressing media market accountability flexibility, professional norm deficiencies, uneven political accountability distribution, and missing public accountability channels.

While these studies have generated valuable insights, significant research gaps persist. Previous investigations have focused on online public opinion or generalized biases rather than examining specific cognitive biases through informatics or communication perspectives. Moreover, minimal research has explored public opinion biases from a field situation perspective, highlighting the need for a comprehensive theoretical framework integrating these perspectives.

Additionally, methodological limitations restrict the practical application of existing research. Qualitative studies have typically emphasized problem analysis and countermeasure development, while quantitative research has primarily employed dynamics models, informatics approaches, and social simulations. The variables in these models tend to be highly academic, producing conclusions that prove difficult to implement operationally.

To address these research gaps, this study integrates perspectives from psychology, informatics, and communication studies to investigate two critical questions: (1) What are the impact mechanisms through which field situation heuristics influence audience cognitive biases during online emergencies? (2) Do demographic differences exist in how field situation heuristics affect cognitive biases, and if so, what patterns emerge across gender, age, and educational background variables?

This research offers both theoretical and practical contributions. Theoretically, it advances the understanding of cognitive bias formation mechanisms in digital environments, contributing to information behavior theory, field theory, and cognitive processing models. Practically, findings will provide evidence-based guidance for government departments and media organizations developing strategies to manage online public opinion, mitigate cognitive biases, and promote healthier information environments.

The paper’s structural arrangement proceeds systematically: The first part establishes research questions based on practical needs and literature analysis. The second part presents research hypotheses and constructs a theoretical model grounded in relevant theoretical foundations. The third part details the research methodology and data collection approach. The fourth part encompasses data processing, model validation, and analysis. The fifth part analyzes, summarizes, and discusses the research results and implications.

### Theoretical basis and research hypothesis

Field theory shows that the field is not merely a physical environment; it also includes the behaviors of others and many factors associated with them (Zinn-Justin, 2021). The field itself influences the cognition and actions of individuals within a field. Meanwhile, situational social theory suggests that humans, as objects of situations, are driven by situations in social interactions (Schneider et al., 2022). In the context of online emergencies, due to the influence of previous similar events on individuals, the priming effect can make users’ cognition of events influenced by the contextual situation, thus facilitating the perception and information processing of similar events in the later stage. Among them, the contextual heuristics people use mainly include anchoring heuristics, representativeness heuristics, and availability heuristics (Jain et al., 2023).

### Situational inspiration and adaptive expectations

Anchoring refers to people’s tendency to connect their evaluation of unknown things with previously adopted cognition. The anchoring effect occurs when people use certain past situations as reference points when evaluating an event. They unconsciously overemphasize the weight of initial information obtained during decision-making, constraining the subject’s cognition and evaluation of the object. The anchoring effect makes people overemphasize prominent and memorable evidence when making judgments, resulting in cognitive biases (Berthet, 2022).

The representativeness heuristics involve judging unknown things based on similar past situations or familiar patterns, with less consideration for the background and environment. There is a tendency to judge the likelihood of an event occurring based on its representativeness or similarity. In this heuristic, the subject mainly draws on past experiences or results of similar things to infer unknown things, often ignoring the basic components of things and leading to cognitive biases (Hjeij and Vilks, 2023). The availability heuristics suggest that information easily retrieved from memory is considered more conventional than information that is not readily remembered. When judging things, people are more likely to base their judgments on how easily certain types of information come to mind (Brashier and Marsh, 2020).

Adaptive expectations refer to people adjusting their psychological expectations for future events based on what happened. It involves mainly using past situations to deduce the future and evaluating and anticipating future situations based on historical results. In the context of online emergencies, situational inspiration in the field of public opinion often leads people to reason, evaluate, and make judgments about events in a fast and convenient way (Wang et al., 2022). The theory of bounded rationality shows that when making decisions, people tend to pursue satisfactory rather than optimal results (Sharot and Sunstein, 2020). Due to limited information acquisition and processing capabilities, people do not collect and process all information when cognizing and evaluating things. Instead, they adjust their psychological expectations for unknown events based on anchoring, representativeness, and available information through convenient pathways and cognitive methods. They use the most obvious and necessary information to make final judgments about events. Based on this, the following hypotheses can be proposed:

*H1a*: In online emergencies, anchoring heuristics inspired by field situations has a significant positive impact on audience adaptive expectations.

*H2a*: In online emergencies, the representativeness heuristics inspired by field situations have a significant positive impact on audience adaptive expectations.

*H3a*: In online emergencies, the availability heuristics inspired by field situations has a significant positive impact on audience adaptive expectations.

### Situational inspiration and implicit attribution

Greenwald and Banaji (1995) introduced the concept of implicit social cognition, a cognitive phenomenon where individuals do not need to deliberately recall or cannot recall an experience. However, this experience potentially significantly impacts their behavior and judgments (Greenwald and Banaji, 1995). Attribution theory points out that in daily interactions, people often intentionally or unintentionally attribute various social phenomena and behaviors in the surrounding environment to understand the development of events and their relationships. This involves associating and deducing the characteristics or behaviors of specific things to seek causal relationships between things or behaviors (Saleh, 2023). Implicit attribution is a deep and complex human cognitive activity during the attribution process. It is a cognitive process where the cognitive subject does not need to deliberately recall to attribute things, and it is an unconscious, subconscious operational process that requires no effort from the cognitive subject (Spatola and Wudarczyk, 2021).

In online emergencies, the priming effect shapes audience cognition through situational inspiration, facilitating the audience’s ability to perceive and process information regarding similar events in the future. The Elaboration Likelihood Model (ELM) suggests that information processing can be divided into central and peripheral routes, with different routes having different persuasive effects on people (Mousavizadeh et al., 2020). The central route shows that after acquiring information, people carefully analyze and reflect on it, distinguishing relevant arguments and searching for clues. Through this route, individuals develop a more nuanced understanding of the information. The peripheral route refers to the tendency of people to identify and judge information content and viewpoints through perceptual cognition after obtaining information. The level of elaboration in information processing is relatively low, leading to quick and intuitive attitude changes. Among them, rough and clues-like information tends to make users more inclined to process and handle information through the peripheral route (Zhou, 2022). The field situation is a collection of all information regarding the subject’s state and the changes in the surrounding environment. It details the state, development trends, and environmental characteristics of things, entities, and users. The situation provides clues-like information. Therefore, when people make causal inferences and judgments among things, the influence of the situation on the subject’s cognition becomes significant. This influence primarily depends on the peripheral route of the ELM model for processing and handling information. Through perceptual cognition, people identify and judge information content and viewpoints, ultimately tending to form subconscious implicit attributions toward things. Based on this, the following hypothesis can be proposed:

*H1b*: In online emergencies, anchoring heuristics inspired by field situations has a significant positive impact on the audience’s implicit attributions.

*H2b*: In online emergencies, representativeness heuristics inspired by field situations have a significant positive impact on the audience’s implicit attributions.

*H3b*: In online emergencies, availability heuristics inspired by field situations have a significant positive impact on the audience’s implicit attributions.

### Expected attribution and cognitive bias

Cognitive bias refers to the distortion phenomenon where people often deviate from objective reality in their cognition of themselves, others, or the external environment due to their own or situational factors (Korteling et al., 2023). Because the first obtained information easily forms an initial impression in people’s cognition, it constitutes the core construct and cognitive schema in perception. The subsequent input information is typically integrated into this memory schema, aligning with the assimilation mode of the previously input information. The follow-up information is implanted quantitatively into the memory framework formed by the preceding information. It causes the subsequently input information to bear the traces and characteristics of the previous information. Therefore, differences in information input order lead to deviations in people’s cognition of things. Among them, the primacy effect is an important way deviation occurs due to information input order. The primacy effect is the influence of the “first impression” formed through the first contact between the subject and object on the subsequent cognition of the object, which is also known as the “preconceived ideas or prejudices” effect. This leads to deviations in the subject’s cognition of similar objects in subsequent cognitions. However, when people cognize and predict unknown events, adaptive expectations mainly adjust their psychological expectations for unknown things based on what has already happened. They use the past results of certain things to predict future situations, and people’s expectations for future situations are mainly based on past historical experience. This can lead to the “preconceived ideas or prejudices” primacy effect in extrapolating things, resulting in cognitive biases (Caballero and Ploner, 2022). Based on this, the following hypothesis can be proposed:

*H4*: In online emergencies, the audience’s adaptive expectations have a significant positive impact on cognitive biases.

When judging the causal relationship of things, Implicit attribution involves unconscious cognitive processes that shape how people explain events and behaviors unconsciously formed by the cognitive subject without deliberate recollection. In the process of attribution, people often commit a fundamental attribution error, which means they tend to attribute others’ behaviors to their inherent traits while ignoring or underestimating the influence of situational factors. Even with sufficient evidence, individuals often underestimate the role of external factors and overestimate the impact of internal factors or personality traits (Mattavelli et al., 2024). This fundamental attribution error generally arises from two main sources. On the one hand, it stems from the motives of the attributing subject, leading to the “actor-observer” effect. Actors tend to attribute positive outcomes to internal factors and negative outcomes to external situations, while observers tend to do the opposite. Observers tend to attribute favorable outcomes to external situations and unfavorable outcomes to internal personal traits. This bias mainly arises from differences in the positions and perspectives of the two parties. Observers often take an idealistic viewpoint, primarily based on conventional logical assumptions. In contrast, actors start from specific situations, emphasizing the unique circumstances that lead to outcomes. On the other hand, attribution bias can also stem from the inherent self-serving tendency in human nature. People tend to attribute others’ favorable and unfavorable outcomes to external factors while attributing others’ unfavorable and favorable outcomes to internal factors. Consequently, in the context of online emergencies, audiences, as observers, tend to have a more pronounced cognitive bias effect when their implicit attributions are stronger due to their perspective as observers combined with the inherent self-serving tendency of human nature. Based on this, the following hypothesis can be proposed:

*H5*: In the context of online emergencies, the audience's implicit attributions have a significant positive influence on cognitive bias.

### Theoretical framework

**Figure 1:**
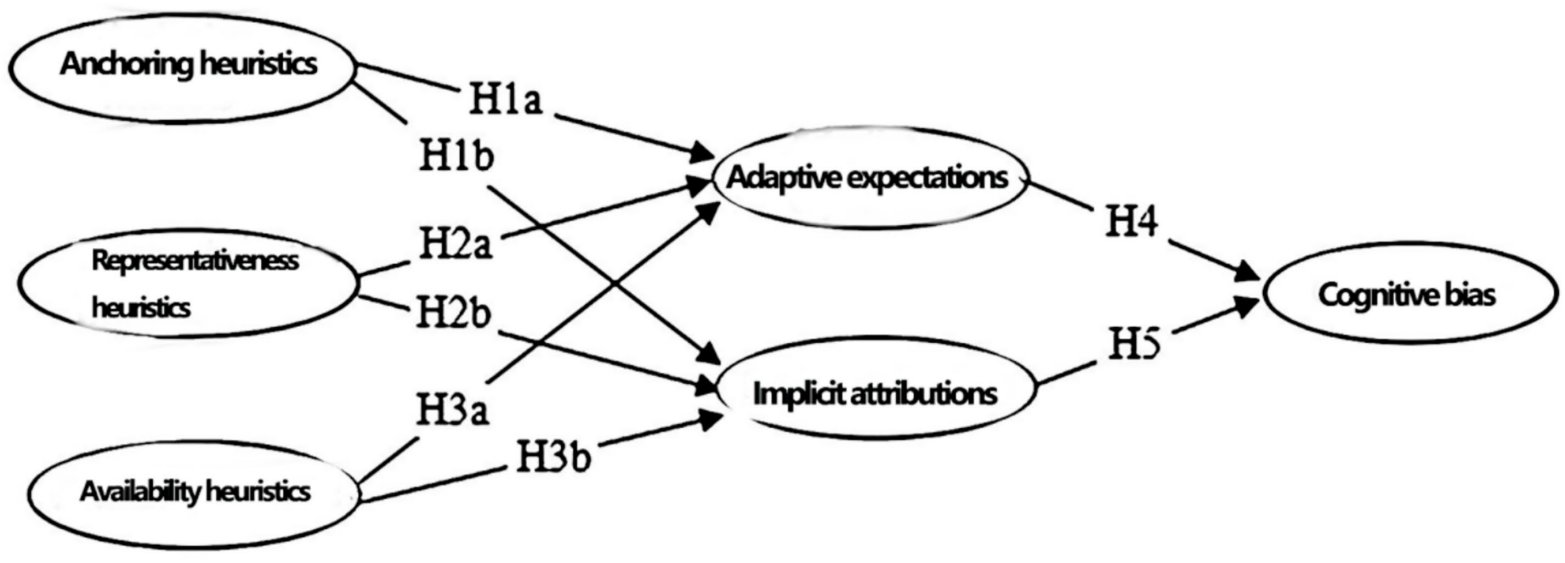
Theoretical framework diagram.

This study takes anchoring heuristics, representativeness heuristics, and availability heuristics in field situations of online emergencies as independent variables, cognitive bias as the dependent variable, and adaptive expectations and implicit attributions as mediating variables. A theoretical model is constructed to investigate how field situation heuristics affect cognitive biases. The theoretical structure is illustrated in Figure 1.

This study posits adaptive expectations and implicit attribution as parallel rather than sequential mediators, grounded in dual-process theory (Kahneman, 2011). Adaptive expectations involve deliberate, conscious adjustment of psychological predictions based on past events—characteristic of System 2 processing. Implicit attribution operates as an automatic, unconscious process requiring no deliberate effort (Greenwald and Banaji, 1995)—characteristic of System 1 processing. These systems function independently and concurrently rather than sequentially (Evans and Stanovich, 2013).

The Elaboration Likelihood Model further supports this distinction: adaptive expectations align with central route processing (deliberate analysis), while implicit attribution aligns with peripheral route processing (heuristic cues). Situational heuristics can simultaneously activate both pathways. Moreover, the two mediators address orthogonal cognitive questions—temporal prediction (“What will happen?”) versus causal explanation (“Why did this happen?”)—which can be engaged concurrently without one preceding the other.

## Method

### Participants and procedure

The data used in this study comes from an online survey conducted during the recruitment period, which started on December 1, 2023, and ended on January 31, 2024, on “The Impact of Situational Context Inspiration in Online Emergency Events on Audience Cognitive Bias.” Several filtering questions were inserted into the questionnaire to enhance the quality and reliability of survey information. Before the formal survey, a pre-survey was conducted with 100 randomly distributed questionnaires to ensure the survey’s reliability and validity. Among them, 81 questionnaires were collected, and after excluding 13 unqualified questionnaires, the final effective response rate was 68%. Reliability and validity analysis showed that the pre-survey questionnaire had a KMO value of 0.87, Bartlett’s test of sphericity *p*-value less than 0.01, a cumulative variance explained of 83.97%, and Cronbach’s *α* value greater than 0.70. In the CITC analysis, the CITC index of Q3 was 0.28, while all other items were greater than 0.50. Therefore, Q3 was removed from the questionnaire, and the remaining items were retained. After deleting Q3, reliability analysis was conducted again on the subscales and the overall scale. The results demonstrated a significant increase in Cronbach’s α value for the subscale that originally included Q3. The Cronbach’s α values for the other subscales and the overall scale were also above 0.70. This indicates that removing Q3 enhanced the optimality of the questionnaire structure, thereby justifying its exclusion.

In practical operation, research team members contacted their friends nationwide via phone, WeChat, face-to-face meetings, and other methods, explaining the survey’s purpose and requirements. They also requested their friends to invite their friends to participate in the survey using the same method. During this process, information such as names, genders, ages, occupations, and educational backgrounds of individuals willing to participate in the survey was recorded and organized. Finally, 2,816 individuals expressed willingness to participate in the survey.

Referring to the demographic distribution characteristics of user populations as of December 2023, from the 53rd “Statistical Report on the Development of the Internet in China” released by the China Internet Network Information Center (CNNIC). A random sampling method combining stratified sampling with multi-stage sampling was adopted using a computer. The first level divided the sampling frame into two groups for random sampling based on the “gender” variable. The second level divided the sampling frame into four groups for random sampling based on the “age group” variable. The third level divided the sampling frame into four groups for random sampling based on the “education level” variable. Finally, 1,000 users were randomly selected as the final survey subjects.

A combination of online survey systems, QQ, WeChat, and other network communication tools was primarily used during the formal survey. To improve the survey’s accuracy and response rate, participants were informed before each survey that they would receive a certain amount of remuneration after completing the survey. The compensation was mainly provided through mobile phone recharge, WeChat red packets, Q coins, Alipay, and other online payment methods.

The data collection process took 2 months, with 1,000 questionnaires distributed and 852 collected. After excluding 96 unqualified questionnaires, 756 valid questionnaires remained, with a response rate of 75.6%. The demographic distribution characteristics of the valid sample data are shown in Table 1.

**Table 1:** Demographic characteristics of the sample (*N* = 756).

Variable	Type	Number	Percentage (%)
Gender	Male	423	55.95%
	Female	333	44.05%
Age	under 30 years old	213	28.13%
	30–39 years old	246	32.59%
	40–49 years old	176	23.26%
	50 years old and above	121	16.02%
Education	College and above	257	33.97%
	High school or vocational school	193	25.57%
	Junior high school	168	22.24%
	Primary or below	138	18.22%
Occupation	Government agencies	105	13.94%
	Public institutions	139	18.45%
	Enterprise groups	278	36.83%
	Self-employed	234	30.78%

Based on the distribution characteristics of demographic variables in Table 1, the sample data covers user groups of different genders, ages, educational backgrounds, and occupations. The sample distribution of various statistical variables does not exhibit extreme or singular cases, making the sample data suitable for research analysis.

### Ethical considerations and informed consent

The Ethics Review Committee of the College of Literature and Journalism at Ocean University of China has exempted this study from ethical approval.

Participants provided informed consent before completing the survey. Consent was obtained in written format through an online system embedded in the survey. The consent process included detailed information about the study’s purpose, procedures, potential risks, benefits, and the voluntary nature of participation. Participants had to acknowledge and agree to the consent form by selecting an option before proceeding to the questionnaire. Since all participants were adults, obtaining consent from parents or guardians was unnecessary. Furthermore, the study did not involve minors.

### Measures

The main variables and item designs for the scales and questionnaires used in this study are as follows:

Dependent variable: cognitive bias refers to the deviation of an individual’s cognition from objective reality due to subjective and objective factors. Based on the research findings of MacLeod and Mathews (2012), with online emergencies as the theme, five measurement items are designed to measure the deviation of audience emotions, attitudes, opinions, and viewpoints from objective facts.

Independent variables: anchoring heuristics refer to the tendency of people to use specific past situations as a starting point when making cognitive judgments about an event and adjust their cognitive results based on this starting point. Referring to the research of Bergman et al. (2010), items are set based on the degree of connection, reference, and evaluation between a specific cue or component and the cognition of unknown things after the audience obtains situational information. There are four items in total.

Representativeness heuristics involves judging unknown events based on past similar situations or familiar cognitive patterns, utilizing previous experiences or outcomes of similar events. Based on the research findings of Teigen (2016), three measurement items are set up according to the degree of reference, degree of reference, and degree of evaluation substitution of representativeness clues or components in the situation to cognize unknown things. Availability heuristics offer an approach for people to judge or process information about unknown things based on certain information that is easily recalled in their minds. Referring primarily to Gigerenzer and Gaissmaier’s (2011) research findings, three measurement items are set based on the degree of connection, reference, and evaluation of clues or components that are easy to notice or recall in the situation.

Mediating variables: adaptive expectations refer to adjusting psychological expectations for future events based on what has already happened, predicting future events’ development based on past events. Referring to Picone et al.’s (2021) research, items are designed based on the degree of reference and borrowing from past events or situations to predict the development of future events. There are four items in total.

Implicit Attribution is a process of unconsciously judging the causality of things without deliberate recall, an unconscious operational process of the cognitive subject. According to Futami et al.’s (2022) research, items are set mainly based on irrationality, automation, and the unconsciousness of users’ judgment of event causality. There are three items in total.

Control variables: in addition to the independent variables affecting the dependent variable, other variables can change the dependent variable’s value in this study. These variables need to be controlled during model construction. If not controlled, both the independent variables and these variables can cause changes in the dependent variable, leading to deviation in research conclusions. Besides social identity, besides the influencing factors explored in this paper, the gender, age, education, and occupation of users are also important factors that affect cognitive bias (Spears, 2021). Therefore, this study sets users’ gender, age, education, and occupation as control variables for their disturbance effects.

Among them, gender is coded as “female” = 1 and “male” = 2. Age is coded as “under 30 years old” = 1, “30–39 years old” = 2, “40–49 years old” = 3, and “50 years old and above” = 4. Education level is coded as “primary or below” = 1, “junior high school” = 2, “high school or vocational school” = 3, and “college and above” = 4. Employment type is coded as “government agencies” = 1, “public institutions” = 2, “enterprise groups” = 3, and “self-employed” = 4.

Except for the control variables, the dependent, independent, and mediating variables are measured using the Likert five-point scale method. Integers from 1 to 5 indicate the degree of agreement with the questions. 1 represents “strongly disagree,” 2 represents “disagree,” 3 represents “uncertain,” 4 represents “agree,” and five represents “strongly agree.”

## Results

### Reliability and validity analysis

#### Scale reliability

The internal consistency of each item in the questionnaire was tested, and the data were processed. The results are shown in Table 2. Among them, the Cronbach’s *α* values for the subscales of anchoring heuristics, representativeness heuristics, availability heuristics, adjustment of expectations, implicit attributions, and cognitive bias are 0.79, 0.74, 0.86, 0.82, 0.74, and 0.85, respectively. The overall Cronbach’s α value for the entire questionnaire is 0.80. All Cronbach’s α values are greater than the 0.70 standard, indicating the reliability of both the subscales and the overall questionnaire design.

**Table 2:** Results of confirmatory factor analysis.

Variable	Indicator	Standard Loading	*t*-value	AVE	CR
Anchoring heuristics	Q1	0.71	13.67	0.73	0.89
	Q2	0.82	5.98		
	Q3	—	—		
	Q4	0.85	12.87		
Representativeness heuristics	Q5	0.74	3.52	0.79	0.92
	Q6	0.76	17.31		
	Q7	0.86	16.13		
Availability heuristics	Q8	0.75	5.92	0.69	0.87
	Q9	0.82	6.84		
	Q10	0.74	7.24		
Adaptive expectations	Q11	0.78	18.94	0.73	0.89
	Q12	—	—		
	Q13	0.73	4.19		
	Q14	0.81	6.57		
Implicit attributions	Q15	0.76	3.24	0.66	0.85
	Q16	0.87	5.44		
	Q17	0.74	16.57		
Cognitive bias	Q18	0.83	20.78	0.80	0.95
	Q19	0.72	14.07		
	Q20	0.87	4.99		
	Q21	0.79	13.23		
	Q22	0.82	4.15		

#### Structural validity

An exploratory factor analysis was conducted on the variables in the scale, and the results are presented in Table 2. When six factors are extracted to represent all the items in the scale, the cumulative variance explained is 85.42%. Meanwhile, the factor loading of item Q12 is 0.38, while the factor loadings of the other items on their respective dimensions are all greater than the standard value of 0.50. Therefore, item Q12 must be deleted, and the remaining items must be retained. These results suggest that the scale has good structural validity in its overall design.

#### Convergent and discriminant validity

A confirmatory factor analysis (CFA) was performed on the collected data, and the results are shown in Table 2. The standardized loading coefficients between each measurement item and the latent variable it measures are greater than 0.70, and their corresponding t-values are greater than the critical value of 1.96 (*p* = 0.05). Additionally, the AVE values of all variables are greater than 0.50, and the composite reliability (CR) values are greater than 0.70. These findings indicate that the observed variables effectively reflect the characteristics of the corresponding latent variables, and there is good consistency among the various observation indicators, demonstrating good convergence of the data. Table 3 shows the correlation coefficients and the square roots of AVE for all latent variables. The square roots of the AVE values for all latent variables are greater than the absolute values of their corresponding correlation coefficients, indicating good discriminant validity among the latent variables.

**Table 3:** Discriminant validity test.

Variable	AH	RH	AH	AE	IA	CB
Anchoring heuristics (AH)	0.85					
Representativeness heuristics (RH)	0.41**	0.89				
Availability heuristics (AH)	0.34*	0.34*	0.83			
Adaptive expectations (AE)	0.54*	0.42**	0.38**	0.85		
Implicit attributions (IA)	0.32**	0.56**	0.48**	0.39**	0.81	
Cognitive bias (CB)	0.49**	0.31*	0.46**	0.34**	0.48**	0.89

The values on the diagonal are correlation coefficients $\sqrt{\mathrm{AVE}}$ , and the remaining values are correlation coefficients. Values marked with * indicate *p* < 0.05, ** indicate *p* < 0.01, and those without a “*” symbol are insignificant at 0.05.

### Path analysis and hypothesis testing

#### Test of causal relationships

For the cognitive bias path model, which involves anchoring heuristics, representativeness heuristics, and availability heuristics, to be valid, it must be based on the significant influence of these heuristics on cognitive bias. Therefore, before analyzing the cognitive bias path model about anchoring, representativeness, and availability heuristics, it is necessary to assess the significance of the causal relationship. The analysis should focus on the relationship between potential exogenous variables and potential endogenous variables. Since all endogenous latent variables are measured using a Likert five-point scale, with variable assignments ranging from “1” to “5,” an ordered Probit model is selected for fitting analysis of the sample data. Additionally, to limit the influence of “control variables,” they need to be included in the equation for analysis.

In Table 4, to ensure parameter identifiability, the parameters have been standardized. The results in Table 4 show that the probability *p*-value of the likelihood ratio chi-square test for the model is 0.000, reaching a significant level of 0.01. This rejects the null hypothesis of an invalid regression model, indicating that the model construction is significantly effective. The quasi-*R*^2^ value corresponding to the model is 0.835, which is relatively large, indicating that the model has good goodness of fit. Meanwhile, the *p*-values of the *z*-test for each coefficient in the model are all less than 0.01, indicating that the estimated values of each coefficient pass the significance test at a 1% confidence level. All coefficients are positive, indicating that anchoring heuristics, representativeness heuristics, and availability heuristics have a significant positive impact on cognitive bias, suggesting a significant causal relationship.

**Table 4:** Ordered Probit model estimation.

Dependent variable	Independent variable		Coefficient	Standard error	*Z*-test value	*p*-value	Model likelihood Ratio Chi-square *p*(chi2) Value	Pseudo *R*^2^
Cognitive bias	Control variables	Gender	0.19**	0.004	9.541	0.000	0.000	0.835
		Age	0.27**	0.052	−2.878	0.004		
		Education level	0.24*	0.045	2.409	0.016		
		Occupation	0.21**	0.048	16.176	0.000		
	Research variables	Anchoring heuristics	0.61**	0.032	5.972	0.000		
		Representativeness heuristics	0.43**	0.034	12.953	0.000		
		Availability heuristics	0.75**	0.028	8.904	0.000		

Values marked with *and ** indicate significance at the 0.05 and 0.01 levels, respectively.

#### Path analysis

**Figure 2:**
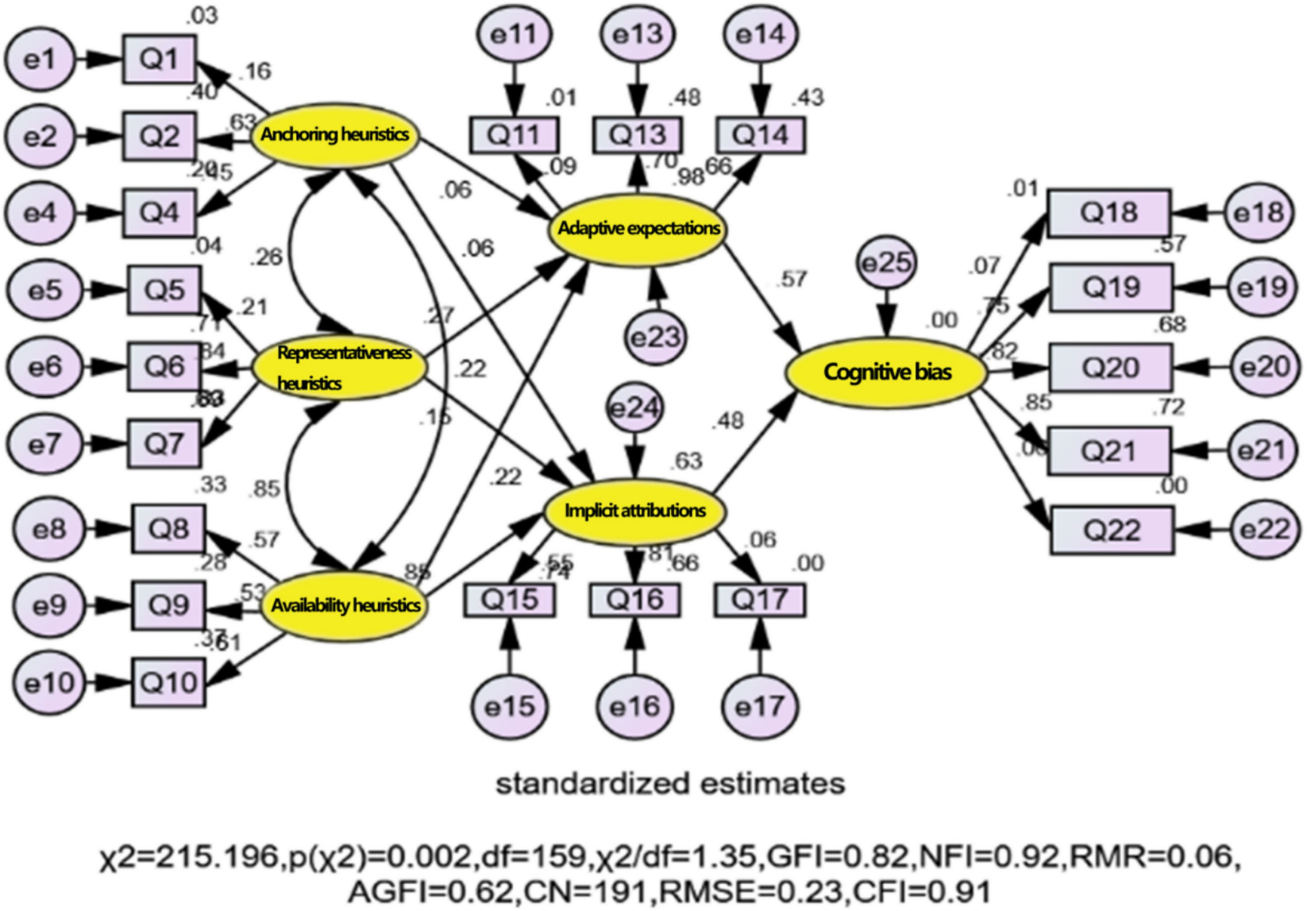
Initial model path fitting effect.

Structural Equation Modeling (SEM) is adopted for path analysis. Since SEM primarily uses the covariance matrix method in data analysis, it can reflect the true relationship and structure between variables. This study employs a complementary three-stage analytical strategy to address the role of demographic control variables. First, the ordered Probit model (Table 4) incorporates all demographic controls (gender, age, education, and occupation) as covariates, establishing that the effects of situational heuristics on cognitive bias are significant after accounting for demographic variation (Pseudo R^2^ = 0.835). Second, the SEM analysis focuses on examining the structural relationships and mediation pathways among the core theoretical constructs without demographic controls, as the primary research objective is to test the theoretical mechanisms rather than to control for demographic confounds, which were already addressed in the first stage. Third, multi-group SEM analysis systematically examines whether the theoretical model operates differently across demographic subgroups (gender, age, and education), providing a more nuanced understanding of demographic influences than simple statistical control (Kline, 2016). This staged approach allows for both rigorous control of demographic variables and detailed examination of how these variables moderate the theoretical relationships, offering complementary insights that a single model with demographic covariates could not provide. Based on the correlation coefficient statistics presented in Table 3, all correlation coefficients between exogenous latent variables are less than 0.40. Conversely, the correlation coefficients between exogenous and endogenous latent variables are greater than 0.50 and significantly correlated at the 0.05 level. This indicates that the variables are suitable for SEM model construction. The theoretical model is estimated using AMOS, and the estimation results are shown in Figure 2.

The fitting results shown in Figure 2 indicate that the model’s goodness-of-fit index, p (χ^2^), is 0.002, below the significance level of 0.05. Therefore, the null hypothesis is rejected. Additionally, while some fit indices such as χ^2^/df = 1.35, NFI = 0.92, and CFI = 0.91 meet the standards, others, including GFI = 0.82, RMR = 0.06, AGFI = 0.62, CN = 191, and RMSE = 0.23 do not meet the criteria for model fit. This suggests that the theoretical model does not align with the observed data, necessitating modifications to the initial model.

**Figure 3:**
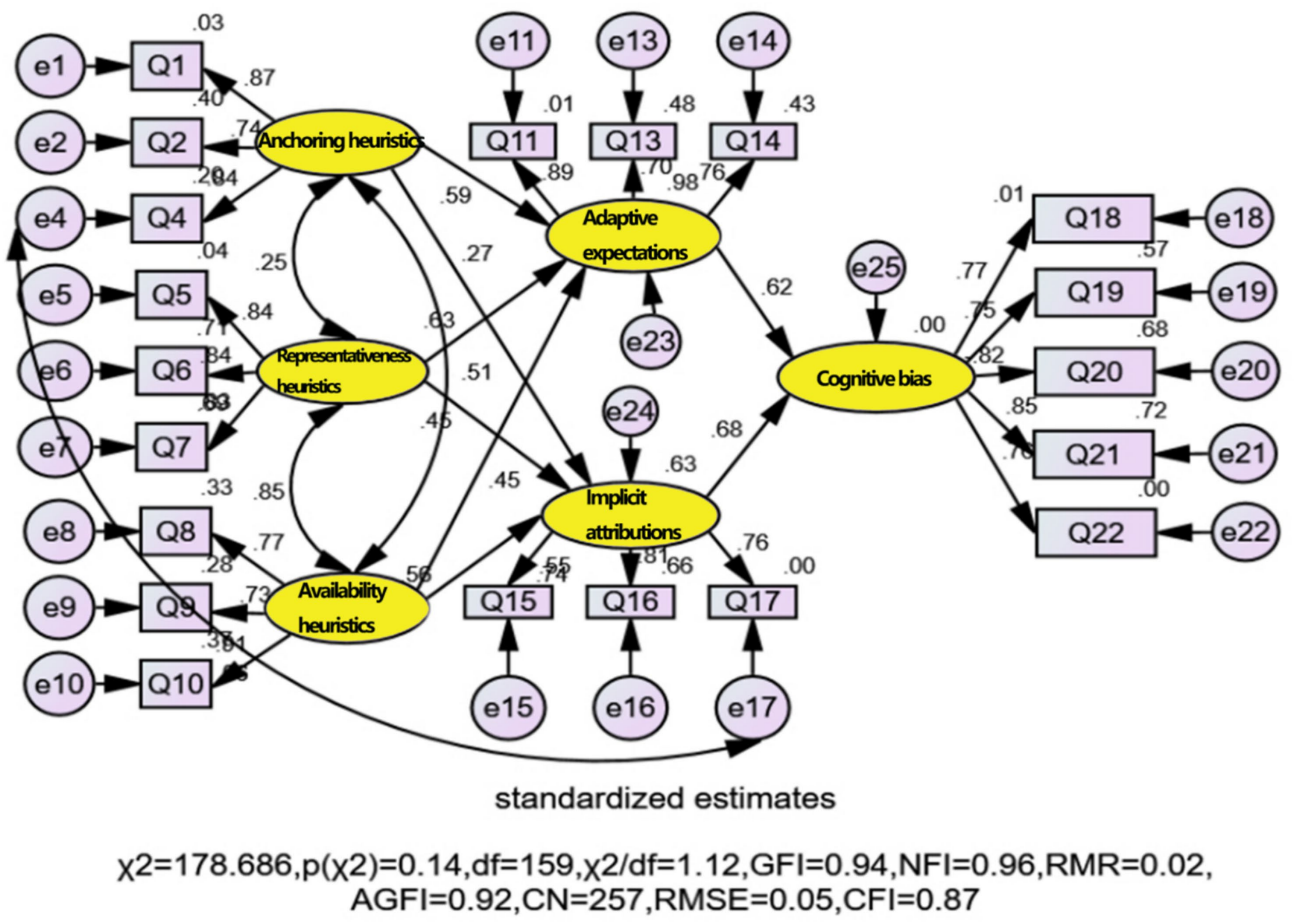
Path fitting effects of the revised model.

Based on the modification indices (M. I.) in the output results, a covariant relationship needs to be established between the error terms of observed variables Q4 and Q17, which can reduce the chi-square value by at least 91.86. This modification is theoretically justified on substantive grounds. Q4 measures the degree to which past situations serve as reference points for evaluating current events (anchoring heuristics), while Q17 assesses the tendency to make implicit causal judgments about events (implicit attribution). Both items share a common methodological feature: they require respondents to engage in retrospective evaluation processes, reflecting on how prior experiences or information influence their current cognitive assessments. This shared method variance, arising from similar retrospective cognitive demands, provides a substantive rationale for allowing their error terms to covary (Kline, 2016). Furthermore, this modification aligns with established SEM practices that permit error covariances when items share systematic variance beyond their latent constructs due to similar wording, measurement timing, or cognitive processes (Byrne, 2016). The modification was applied conservatively, adding only one error covariance based on both statistical criteria (highest M. I. value) and theoretical justification, rather than making multiple atheoretical modifications. Consequently, a covariant relationship is established between the error variables of measurement indicators Q4 and Q17, forming the initially modified model, which is then estimated. The estimation results are shown in Figure 3.

The fitting results shown in Figure 3 indicate that the various goodness-of-fit indices for the modified model are as follows: p(χ2) is 0.14, greater than 0.05. Therefore, the null hypothesis cannot be rejected, suggesting that the modified model aligns well with the sample data. Simultaneously, among the various fit indices, except for the CFI value of 0.87, which fails to meet the standard, the other indices, including χ2/df = 1.12, GFI = 0.94, RMR = 0.02, AGFI = 0.92, CN = 257, RMSE = 0.05, and NFI = 0.96, all meet the criteria for model fit. Additionally, there are no parameters in the output of the modification indices that require further correction, indicating that the settings of the modified model are acceptable. Please refer to Table 5 for the fitting results of each path coefficient and significance.

**Table 5:** Fitting results of the revised model.

Paths	Regression weights				Standardized regression weights
	Estimate	S. E.	C. R.	P	Estimate
Adaptive expectations ←Anchoring heuristics	0.500	0.031	−9.673	***	0.591
Implicit attributions ← Anchoring heuristics	0.152	0.042	14.621	***	0.268
Adaptive expectations ← Representativeness heuristics	0.622	0.025	7.412	***	0.632
Implicit attributions ← Representativeness heuristics	0.627	0.063	17.841	***	0.514
Adaptive expectations ← Availability heuristics	0.792	0.036	5.399	***	0.449
Implicit attributions ← Availability heuristics	1.289	0.073	17.556	***	0.562
Cognitive bias ← Adaptive expectations	1.026	0.059	17.503	***	0.618
Cognitive bias ← Implicit attributions	1.004	0.039	25.508	***	0.684

***significance level (*p* < 0.001).

**Figure 4:**
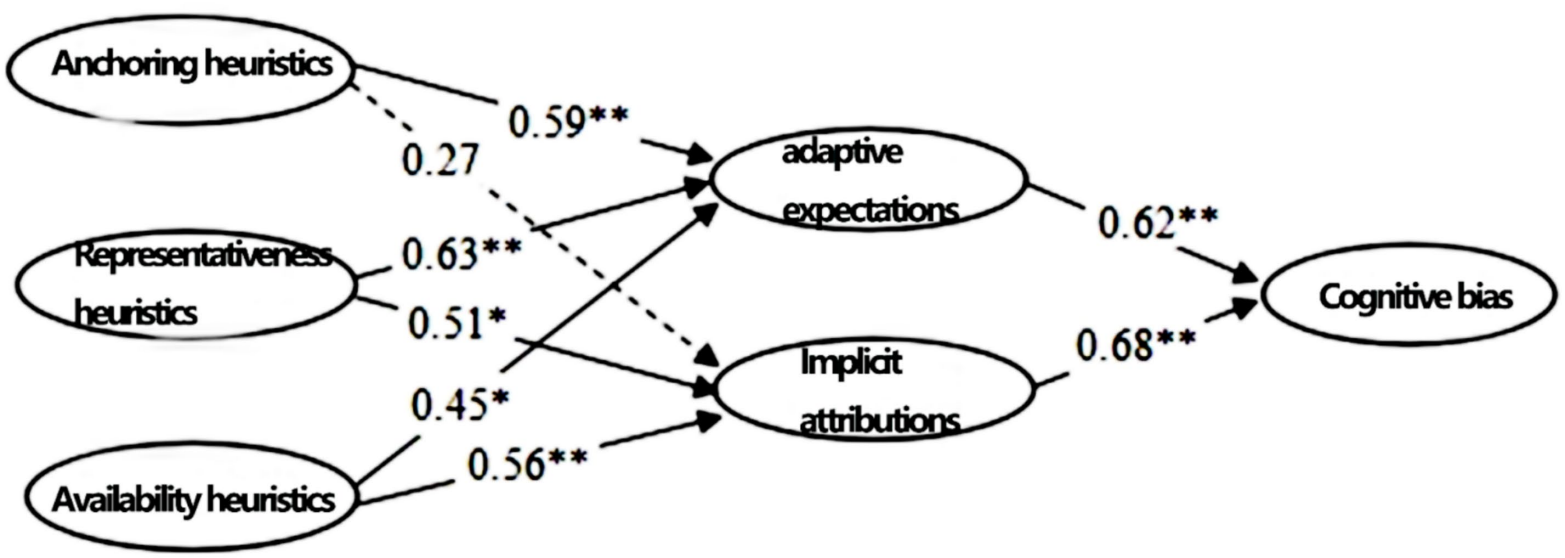
Path coefficient diagram of the model n*ote:* * indicates *p* < 0.05, ** indicates *p* < 0.01, and values without a “*” are insignificant.

The fitting results in Table 5 show that the standardized regression weights of the paths are all between 0 and 1. The standardized loads of each observed variable on the corresponding latent variable are greater than the standard value of 0.70. Additionally, all path coefficients pass the significance test at the 0.05 significance level, indicating that the parameter estimation results of the modified model are acceptable. Based on the parameter fitting results, the standardized path coefficients and significance corresponding to the main effect paths of the theoretical model are shown in Figure 4.

Based on the standardized path coefficient diagram in Figure 4, it can be seen that the absolute values of all path coefficients are between 0 and 1. Except for H1b, whose corresponding *t*-test did not reach the 0.05 significance level, all other hypotheses’ *t*-tests achieved the 0.05 significance level. According to the positivity or negativity of each standardized path coefficient and the results of the *t*-tests, it is indicated that apart from H1b, which did not receive support, the other seven hypotheses all received empirical support. Meanwhile, according to the magnitude of each path coefficient, anchoring, and representativeness heuristics have a greater impact on adaptive expectations (with path coefficients of 0.59 and 0.63, respectively) compared to implicit attributions (with path coefficients of 0 (taken as 0 if not significant) and 0.51, respectively). The availability heuristics have a greater impact on implicit attributions (with a path coefficient of 0.56) than adaptive expectations (with a path coefficient of 0.45). Implicit attributions have a greater impact on cognitive bias than adaptive expectations (with path coefficients of 0.68 and 0.62, respectively).

#### Mediating effect

Based on the mediating effect analysis procedure proposed by Qin (2024), combined with the Bootstrap method proposed by Hayes (2017) for mediating effect testing. The structural setup of the model was conducted with cognitive bias as the dependent variable. Anchoring heuristics, representativeness heuristics, and availability heuristics were used as independent variables and adaptive expectations and implicit attributions served as mediators. Model 1 from the Process program was selected, and 1,000 repeated sample extractions were performed, with a 95% confidence interval for significance in mediating effect testing. The test results are shown in Table 6.

**Table 6:** Results of the mediating effect test.

Mediation Path	Effect Size	Standard Error	LLCI	ULCI
Anchoring heuristics → Adaptive expectations → Cognitive bias	0.2258	0.0331	0.1611	0.2905
Anchoring heuristics → Implicit attributions → Cognitive bias	0.0436	0.0328	−0.0191	0.1063
Representativeness heuristics → Adaptive expectations → Cognitive bias	0.2506	0.0316	0.1898	0.3114
Representativeness heuristics → Implicit attributions → Cognitive bias	0.2068	0.0323	0.1441	0.2695
Availability Heuristics → Adaptive Expectations → Cognitive Bias	0.1390	0.0319	0.0782	0.1998
Availability heuristics → Implicit attributions → Cognitive bias	0.2408	0.0334	0.1761	0.3055

In Table 6, if the confidence interval’s lower limit (LLCI) and upper limit (ULCI) do not include 0, they indicate a significant mediating effect; otherwise, the mediating effect is insignificant. The calculation results in Table 5 show that among the tested mediation paths, only the path “Anchoring Heuristics → Implicit Attributions → Cognitive Bias” has a 95% confidence interval that includes 0. All other paths have confidence intervals that do not include 0, indicating that apart from the “Anchoring Heuristics → Implicit Attributions → Cognitive Bias” path, which has an insignificant mediating effect, all other paths exhibit significant mediating effects.

Based on the estimated mediating effects, the total effects of anchoring heuristics, representativeness heuristics, and availability heuristics on Cognitive Bias through the two mediators of Adaptive Expectations and Implicit Attributions are 0.2258, 0.4574, and 0.3798, respectively. Specifically, anchoring heuristics have a more significant impact on cognitive bias through the mediator of adaptive expectations (with a mediating effect coefficient of 0.2258) compared to implicit attributions (whose coefficient is not significant, resulting in a mediating effect of 0); representativeness heuristics have a stronger influence through adaptive expectations (mediating effect coefficient of 0.2506) than through implicit attributions (mediating effect coefficient of 0.2068); availability heuristics affect cognitive bias more significantly through implicit attributions (mediating effect coefficient of 0.2408) than through adaptive expectations (mediating effect coefficient of 0.1390).

#### Test results

Based on the above test results, a summary of the hypothesis testing results is provided in Table 7.

**Table 7:** Hypothesis test results.

Hypothesis	Path	Standardized Coefficient	*t*-value	Test result
H1a:+	Anchoring heuristics → Adaptive expectations	0.59	16.83	Support
H1b:+	Anchoring heuristics → Implicit attributions	0.27	1.61	Not Supported
H2a:+	Representativeness heuristics → Adaptive expectations	0.63	6.97	Support
H2b:+	Representativeness heuristics → Implicit attributions	0.51	2.89	Support
H3a:+	Availability heuristics → Adaptive expectations	0.45	2.14	Support
H3b:+	Availability heuristic → Implicit attributions	0.56	4.83	Support
H4:+	Adaptive expectations → Cognitive bias	0.62	7.25	Support
H5:+	Implicit attributions → Cognitive bias	0.68	21.62	Support

Table 7 shows that the path coefficients corresponding to each hypothesis are between 0 and 1 among all the research hypotheses. Except for H1b, which has a |*t*| value of 1.61 (less than the critical value of 1.96 corresponding to a significance level of 0.05), the |*t*| values of the significance tests for all other coefficients are greater than 1.96. This indicates that apart from H1b, which is not supported by the data, all other research hypotheses are supported.

#### Group analysis

According to the Information Environment Usage Theory, users’ occupations and social roles significantly influence their information behavior, shaping distinct characteristics in their information-seeking patterns. We analyzed differences across demographic groups and analyses based on gender, age, and educational background. It should follow the validation of the theoretical framework mentioned above.

During the estimation of the group model, most of the goodness-of-fit indices met the standard values, except for the AGFI value (0.79) in the gender group and the NFI value (0.86) in the age group. This suggests that the group data for gender, age, and educational background generally fit well with the theoretical model. All standard path coefficients fall within the range of 0 to 1, and the *t-*tests for each corresponding coefficient reach a significance level of 0.05. This indicates that the hypothetical theoretical model is valid across gender and age groups. The analysis results are summarized in Table 8.

**Table 8:** Group analysis results.

Path	Standardized coefficient							
	Gender		Age group			Education level		
	Female	Male	50+	30 ~ 50	30-	Primary or below	Middle-High School	College or above
Anchoring heuristics → Adaptive expectations	0.63	0.48	0.45	0.55	0.65	0.68	0.48	0.44
Anchoring heuristics → Implicit attributions	—	—	—	—	—	—	—	—
Representativeness heuristics → Adaptive expectations	0.67	0.51	0.48	0.58	0.69	0.73	0.51	0.48
Representativeness heuristics → Implicit attributions	0.55	0.41	0.39	0.47	0.56	0.59	0.42	0.38
Availability heuristics → Adaptive expectations	0.48	0.36	0.35	0.42	0.50	0.52	0.38	0.33
Availability heuristics → Implicit attributions	0.60	0.45	0.43	0.52	0.62	0.64	0.46	0.42
Adaptive expectations → Cognitive bias	0.66	0.50	0.48	0.57	0.68	0.71	0.51	0.47
Implicit attributions → Cognitive bias	0.73	0.55	0.52	0.63	0.75	0.78	0.55	0.52

Based on Table 8, the order of path effects for gender groups is consistently females vs. males. For age groups, the order of path effects is “under 30 years old,” “30 to 50 years old,” and “50 years old and above.” For education groups, the order of path effects is “Primary or below,” “Middle-High School,” and “College or above.”

## Discussion

### Summary of findings

The empirical findings validate the proposed theoretical framework examining how situational heuristics influence cognitive biases in online emergencies. The structural equation modeling results demonstrate good model fit (χ2/df = 1.12, GFI = 0.94, RMR = 0.02, AGFI = 0.92), indicating that field situational heuristics significantly affect cognitive biases through adaptive expectations and implicit attributions. This integrated model explains 83.5% of the variance in cognitive biases, suggesting its strong explanatory power for understanding bias formation in online emergency contexts.

Among all the hypotheses on the path of structural equation modeling, H1b was not supported, indicating that the anchoring heuristics in situational context do not significantly affect the audience’s implicit attributions. This result may be due to the anchoring effect, which leads people to associate unknown judgments with past cognitive experiences. It causes individuals to use a past specific situation as a reference point when evaluating an event. They unconsciously emphasize initial information during decision-making, thus constraining their cognition and evaluation of objects. In uncertain situations, anchoring heuristics leads people to use a certain reference point to reduce the cognitive process’s ambiguity and then make varying degrees of adjustments to reach a conclusion (Doyle et al., 2021). However, implicit attribution, as a deep and complex human cognitive activity, is a cognitive process where the cognitive subject unconsciously attributes things without deliberate recollection. It is a subconscious operational process that requires no effort from the cognitive subject (Petty and Cacioppo, 1986). Therefore, since anchoring heuristics rely on reference points for cognitive adjustment before forming a conclusion, activating the previously formed stable implicit cognition and attribution is difficult. Hence, in empirical testing, the impact of anchoring heuristics on users’ implicit attribution appears insignificant.

The effects of anchoring heuristics, representativeness heuristics, and availability heuristics on cognitive biases exhibit differences based on demographic variables in user group analysis. This variation may primarily stem from the diverse social characteristics and roles among different groups. General information behavior theory suggests that users’ occupations and social roles significantly impact their information behavior. This cultivates distinct characteristics in their information-seeking patterns.

Additionally, numerous intermediary variables influence information behavior, including psychological traits, demographic features, and social roles. These factors affect dynamics during information acquisition and utilization. Regarding our study’s findings, gender differences are not only influenced by physiological factors but also, more significantly, by sociocultural gender disparities. Firstly, due to differences in social status and roles between men and women and society’s differing expectations and evaluations of different genders, there are a series of distinct characteristics in terms of behavioral norms and gender stratification. Therefore, the physiological differences between genders in a group, influenced by social norms, the power of social institutions, and the subtle impact of gender culture accumulated over generations, have led to differences in information processing patterns and information behaviors between men and women (Oeberst and Imhoff, 2023). Secondly, regarding user age, age is often closely related to a person’s physiological and intellectual development. It also represents the richness of their social experience and reflects differences in the maturity of their thinking. At the same time, influenced by social norms, social culture, and customs, groups of different age stages play different social roles and bear different social responsibilities, which have an important impact on the information-thinking mode, cognitive style, and behavioral characteristics of individuals of different ages. Finally, regarding users’ educational level, since education and learning are important ways for people to change and reshape their thinking and cognitive styles through acquired efforts, the level of education reflects the differences in the degree of education individuals receive in society. Those with higher education improve their cognition and attitude toward things through learning more scientific knowledge and more formal training. Compared with those with lower education, they have more cautious and scientific thinking and cognition. Hence, individuals with different educational backgrounds usually have different degrees of differences in their cognition, attitude, and behavior toward things.

Regarding the significant influence of contextual heuristics on cognitive bias through the mediating effect of adapting expectations and implicit attribution, although there has not been any research literature that is completely consistent with this research conclusion in the past, this research conclusion is consistent with the deductive conclusions of past related research. The “Heuristic-Systematic Model” (HSM) points out that information cognition can be divided into heuristic and systematic based on people’s differences in the level of effort put into information processing. Different methods will have different persuasive effects on people. Among them, systematic processing involves comprehensive and careful consideration of both explicit and implicit cues in information, and attitudes and responses are formed based on this. This approach emphasizes careful analysis and reflection on information after acquiring it, actively screening relevant arguments, and carefully searching for relevant clues. The subject’s cognition of information in this path is more precise. Heuristic processing, in contrast, processes and handles information mainly based on the principle of “minimum cognitive effort.” People tend to identify and judge information content and viewpoints through perceptual cognition, and the level of precision in information processing is relatively low. Thus, the subject quickly and intuitively forms attitudes and viewpoints. Among them, rough clue information tends to make users more inclined to process and handle information in a heuristic way. For clue-based contextual information, such as anchoring information, representative information, and availability information, due to the limited use of information by the subject, people tend to adopt a heuristic approach to process the information, identifying and judging the content through perceptual cognition, which can easily lead to cognitive biases about things (Dror, 2020).

### Theoretical implications

This study offers significant theoretical contributions to understanding cognitive bias formation in the digital media environment. By integrating field theory with heuristic information processing theory, we establish that situational heuristics in online emergencies influence cognitive biases through dual mediating pathways of adaptive expectations and implicit attributions. This theoretical framework extends beyond traditional individual-psychological perspectives to consider the structural influence of the media environment. The differentiated effects of representativeness, availability, and anchoring heuristics reveal the complex interplay between contextual factors and cognitive processes in digital spaces. Furthermore, the demographic variations in heuristic effects highlight the socio-cultural dimensions of cognitive bias formation, contributing to a more nuanced understanding of audience behavior in networked communication environments. These insights advance media psychology and digital communication theories by illuminating how environmental factors shape cognitive outcomes in online public opinion formation.

### Practical implications

The main findings of this study indicate that, in online emergencies, contextual heuristics (including anchoring heuristics, representativeness heuristics, and availability heuristics) significantly influence cognitive biases through the mediating effects of adaptive expectations and implicit attribution. To better leverage these findings, we propose several actionable recommendations for managing online emergencies:

Strengthen Online Public Opinion Management: The results of this study demonstrate that the impact of anchoring heuristics, representativeness heuristics, and availability heuristics on cognitive biases should not be underestimated. Therefore, when dealing with online emergencies, managers should focus on minimizing the influence of these contextual heuristics, especially during the initial stage of information dissemination. To counter cognitive biases, it is recommended to reduce anchoring effects by providing more transparent and comprehensive information while enhancing the diversity of information sources to avoid bias propagation caused by representativeness and availability heuristics.

Develop Targeted Information Dissemination Strategies: In emergencies, the speed and breadth of information dissemination significantly impact public cognition. According to the findings of this study, adaptive expectations and implicit attribution significantly affect the formation of cognitive biases. Therefore, policymakers can utilize this insight to design targeted communication strategies that guide the public in avoiding bias formation during information release. For example, in the early stages of a crisis, the government can release authoritative statements and expert opinions through the media to help the public avoid unreasonable attributions and over-reliance on initial information in making judgments.

Use Education and Training to Reduce Bias: To fundamentally reduce the negative impact of cognitive biases, it is recommended that public education and psychological intervention measures be implemented. These measures should include raising public awareness of cognitive biases and information processing methods, helping them recognize and avoid the excessive influence of contextual heuristics, particularly during emergencies and online crises. Additionally, educational content can incorporate the psychological mechanisms of adaptive expectations and implicit attribution, helping the public cultivate more rational information processing habits.

### Limitations

Although this study has tried to improve the research design as much as possible, there are still some limitations due to objective conditions. In data collection, although the sampling design and various processes and details of data collection have been improved as much as possible, and the scope of data acquisition has been expanded to the maximum extent possible, data collection requires a lot of manpower and material resources. However, due to the limitations of the research team’s manpower and material resources, there is still room for improvement in this study. In online sampling surveys, respondents are concerned that the surveyors may have malicious intent or be probing their privacy, resulting in a low response rate. Therefore, in subsequent related studies, sampling surveys can be considered among real people to improve the questionnaire’s response rate and qualification rate. Additionally, while our study examined cognitive biases across four types of online emergencies (public safety, social conflict, corporate misconduct, and government action), future research should explore whether the effects of situational heuristics vary significantly across these different types. The current study provided participants with examples from each category but did not analyze the potential differences in cognitive mechanisms between these distinct types of Online Emergencies. Different emergency types might trigger varying levels of emotional involvement, personal relevance, and prior knowledge, potentially influencing how situational heuristics affect cognitive biases.

## Conclusion

In conclusion, this study demonstrates the significant impact of situational heuristics on cognitive biases during online emergencies. The findings underscore the importance of considering both adaptive expectations and implicit attributions in shaping public opinion and decision-making in crises. Future research should explore these dynamics in diverse contexts and populations, potentially incorporating advanced technologies like big data and artificial intelligence to better manage information flow and mitigate cognitive biases in real-time crisis scenarios.

## Data Availability

The raw data supporting the conclusions of this article will be made available by the authors, without undue reservation.
